# Assessment of Postpartum Depression in Adolescents Who Delivered during COVID-19 Social Restrictions: The Experience of a Tertiary Hospital from Bucharest, Romania

**DOI:** 10.3390/healthcare9070807

**Published:** 2021-06-26

**Authors:** Alexandra Matei, Mihai Cornel Traian Dimitriu, Catalin Gabriel Cirstoveanu, Bogdan Socea, Cringu Antoniu Ionescu

**Affiliations:** 1 Department of Obstetrics and Gynecology, Carol Davila University of Medicine and Pharmacy Doctoral School, 020021 Bucharest, Romania; cringu.ionescu@umfcd.ro; 2 Department of Obstetrics and Gynecology, Sf. Pantelimon Emergency Clinical Hospital, 021659 Bucharest, Romania; mihai.dimitriu@umfcd.ro; 3 Department of Pediatrics, Carol Davila University of Medicine and Pharmacy, 020021 Bucharest, Romania; catalin.cirstoveanu@umfcd.ro; 4 Department of Surgery, Carol Davila University of Medicine and Pharmacy, 020021 Bucharest, Romania

**Keywords:** adolescent pregnancy, postpartum depression, COVID-19, pandemic, confinement

## Abstract

In the context of the viral spread of COVID-19 in 2020, Romanian authorities declared national confinement for two months. Our country faces a public health issue regarding adolescent pregnancy. This study assessed the predisposition of teenage mothers to postpartum depression and the influence of the viral pandemic on their emotional status. This study enrolled patients 10 to 19 years old who delivered in our department between March–December 2020. Teenagers were attributed to the “lockdown group” (*n* = 30) and the “open group” (*n* = 171). All study participants agreed to take an interview based on a three-part questionnaire, including the Edinburgh Postnatal Depression Scale (EPDS). In the “lockdown group”, 16.67% of patients felt stressed over the last year compared to 11.11% of individuals in the “open group”, but there was no statistically significant difference between groups regarding overall EPDS scores (z value 0.51, Mann–Whitney U test). Predictable variables for postpartum depression were the use of cigarettes (OR = 1.08, 95% CI: 1.00–1.16), intended pregnancies (OR = 0.25, 95% CI: 0.09–0.68, *p* = 0.007) and absence of stressors in the last year (OR = 0.07, 95% CI: 0.02–0.30, *p* = 0.0002). More adolescents were stressed during confinement compared to those who delivered in the following time period; this aspect did not interfere with depression screening scores. A planned pregnancy, even during adolescence, can serve as a protective factor for postpartum depression.

## 1. Introduction

From the beginning of the COVID-19 pandemic until the end of 2020, official reports identified a total number of 632,263 infected persons on Romanian territory, of whom 101,054 cases belonged to Bucharest capital city [1]. Multiple measures were established to decrease viral spread, especially in medical units. Of these, prohibited access of visitors in maternity wards along with limited interaction of patients with caregivers heightened the risk of postpartum depression [2,3,4]. The World Health Organization (WHO) addressed a new statement on mental health among adolescents, with particular attention given to teenagers exposed to adversity like those experiencing adolescent pregnancy [5].

Romania ranks second in the European Union after Bulgaria regarding adolescent pregnancies, with 16,639 cases officially registered in 2019 [6]. Screening for peripartum depression—or any other mental condition that can occur perinatally—is not performed in our country, regardless of the age of the mother. Due to its associated social, healthcare expenses and lack of clear standard practice in the medical field of reproductive health, researchers involved in conducting this study aimed to raise awareness of this national issue and its long-term consequences. 

Studies have shown that untreated depression during pregnancy can lead to preterm birth, preeclampsia, low birth weight, while during the postpartum period, it can trigger maternal anxiety, failure to initiate breastfeeding, and absence of maternal bonding with the infant; in severe cases, it can determine alterations in fetal grey matter or even suicide or infanticide [7,8]. The main point of interest in this study has been the screening for symptoms of emotional distress in teenagers during the postpartum period. Patients were asked to respond to a short interview, including the Edinburgh Postnatal Depression Scale (EPDS), which is a validated screening tool for perinatal depression in the Romanian population [9,10]. In the COVID-19 pandemic context, additional questions on external stressors have also been introduced in the survey, as we considered it essential to determine whether social restrictions would make pregnant adolescents more vulnerable and prone to postpartum depression, as cited in similar studies [11].

## 2. Materials and Methods

Department of Obstetrics and Gynaecology of “St. Pantelimon” Emergency Hospital is a tertiary medical unit delivering approximately 1600 babies annually from Bucharest and surrounding areas. During the Covid-19 pandemic, our hospital has been a non-designated emergency unit for treating positive patients; individuals with clinical suspicion and those who tested positive for COVID-19 using molecular diagnosis were transferred to designated medical units for birth assistance. 

We have conducted a prospective study to assess the predisposition of adolescent mothers to postpartum depression and the influence of the COVID-19 pandemic on their emotional status. 

This analysis was possible with hospital ethics committee approval (2134/21.01.2020). All procedures performed in this study were in accordance with the ethical standards of the institutional ethics committee and with the 1964 Helsinki declaration and its later amendments or comparable ethical standards. 

Pregnant patients aged ≤ 19 years old who gave birth in our clinic from 16 March 2020 to 31 December 2020 and who agreed to fill in our questionnaire were enrolled in our study. Exclusion criteria consisted of patients aged 20 years old or more and those who did not accept to complete our survey. There were no COVID-19 positive patients included in this study. 

Obstetricians conducted the interviews following a structured questionnaire template that included three parts: (I) Demographic and social data, (II) Obstetrical history, and (III) EPDS. The first part encompasses questions about social development, education: “How many years of school have you finished?” and “Can you read and write?”; family background: “Do you have family support?”, “In your family, is it a tradition having a child during adolescence?”; emotional status: “If you could change the past, would you change the moment you became pregnant and gave birth?” and “Did you feel stresses over the past year?”. All answers were anonymous. Informed consent was obtained from all participants included in the study or their parents. The poor hospital addressability and follow-up of teenagers during pregnancy determined researchers to complete the questionnaires on day 2 or 3 postpartum, during patient hospitalization. Obstetrical data were obtained from each patient’s electronic medical charts and from birth registries. 

The EPDS is a 10-item screening tool for perinatal depression, which can be utilized both in the antenatal and postnatal period; questions are scored 0 to 3 points and then summed up with possible final scores between 0 and 30. A cut-off value for “possible depression” was set above 10. Patients scoring above 13 and those who responded positively in question 10 concerning suicidal thoughts were considered suggestive for referral.

COVID-19 epidemiological background determined Romanian authorities to establish a state of national emergency on 16 March 2020; it was on 15 May 2020 that interdictions were removed and people exit lockdown. These national regulations have been considered in our analysis as possible external additional stressor factors, and their influence on pregnant teenager’s emotional status was sought. In this context, we have divided our study population into 2 groups: one including patients who delivered during the confinement period (lockdown group): 16 March to 15 May 2020, and the second group including patients who delivered in the following period (open group): 16 May to 31 December 2020. Examining patients separately depending on the restrictive norms applied in our country should help anticipate the degree to which social regulations manage to impact adolescent compliance to the birth process additionally to other transformative teenage events. It also gives insight for physicians on how to address this population of pregnant women, both obstetrically and psychologically. 

Based on EPDS corresponding scores and the cut-off values mentioned in this section, patients were divided into 3 categories as follows: scores ≤10 points were considered normal (N); scores 11–13 points were suspected of postnatal depression (S); scores above 13 and positive response in question 10 was considered very suspicious (VS) and suggestive for referral. We considered this division to be effective in identifying young mothers at risk of developing perinatal depression and in identifying any possible age-related correlation. 

Statistical analysis of data was performed using NCSS Data Analysis 2020, NCSS, LLC, Kaysville, Utah, USA. Descriptive statistics of continuous variables identified means and standard deviations; frequencies were identified for categorical variables. Multinomial logistic regression analysis was performed on all datasets, considering EPDS score as categorical dependent variable: normal (N), suspected (S), and very suspicious (VS). Numeric independent variables included in the test were age, education, number of sexual partners, parity, abortions (induced and spontaneous), number of smoked cigarettes per day; categorical (binomial: “yes”/”no”) independent variables were family support, presence of stressors over the last year and unintended pregnancy. Relevant results were expressed in odds ratios (OR) with 95% confidence intervals (95% CI). Mann–Whitney U test was performed to identify any significant differences between overall EPDS scores and gestational age in the two study groups: lockdown and open; *p*-value < 0.05 was considered statistically significant. 

## 3. Results

There were 207 pregnant adolescents eligible for the analysis that gave birth in our clinic during the study period. Six of them refused to take the interview and were, therefore, excluded from the report. A total of 201 patients remained in the study and were divided into two categories based on the time of delivery: the lockdown group, including patients who gave birth during confinement (*n* = 30), and the open group, including those who delivered in the forthcoming period (*n* = 171). 

Demographic and social variables are presented in Table 1. Patients were aged 17.4 +/± 1.3 years (range 14–19 years). In our report, we enrolled 52.74% pregnant girls in their late adolescence: 17 to 19 years old, and 2.99% in early adolescence: aged 10 to 14 years old. None of the early adolescents gave birth during the confinement. Most participants came from rural areas (73.63%) and finished between five and eight years of school (52.24%). There were 19 patients (9.45%) who never went to school, 13 of them (68.42%) aged 17 to 19 years old (“How many years of school have you finished?”). Overall, most of the patients lived in concubinage (8.59%) and had family support (95.02%) (“Do you have family support?”). Results showed that 85.07% of pregnancies were intended (“If you could change the past, would you change the moment you became pregnant and gave birth?”) and that happened because of family tradition in most of the cases (overall: 66.67%, the lockdown group: 66.67%, and the open group: 66.67%) (“In your family, is it a tradition having a child during adolescence?”). A higher proportion of patients from the lockdown group felt stressed over the last year compared to individuals in the open group: 16.67% versus 11.11% (“Did you feel stressed over the past year?”).

Teenagers had similar mean gestational age in both study groups: 37.9 ± 1.82 weeks in the lockdown group and 38.05 ± 2.05 weeks in the open group, as shown in Table 2.

Statistical tests performed on EPDS scores provided relevant information on possible predictors of postpartum depression (Table 3). Regression coefficients indicate that the number of cigarettes used per day as a numeric predictor can significantly discriminate between teenagers in the EPDS S group and those in the EPDS N group (b(i) = 0.08, Sb(i) = 0.03, *p* = 0.03); the higher the number of used cigarettes per day in a patient, the more probable the patient is to obtain an EPDS S category score (OR = 1.08, 95% CI: 1.00–1.16, *p* < 0.05). The same regression model identified ‘stressed over the last year = no’ as a protective factor for obtaining EPDS vs. scores and developing postpartum depression: OR = 0.07, 95% CI: 0.02–0.30, *p* = 0.0002. ‘Unintended pregnancy = no’ was also a significant predictor for both EPDS S and vs. scores (OR = 0.25, 95% CI: 0.09–0.68, *p* = 0.007 and OR = 0.10, 95% CI: 0.02–0.45, *p* = 0.002), patients having lower likelihood of developing postpartum depression. Adolescents obtaining EPDS S scores were correctly predicted by the model in 45.16% of the cases and those scoring EPDS vs. in 70.58% of the cases.

**Figure 1:**
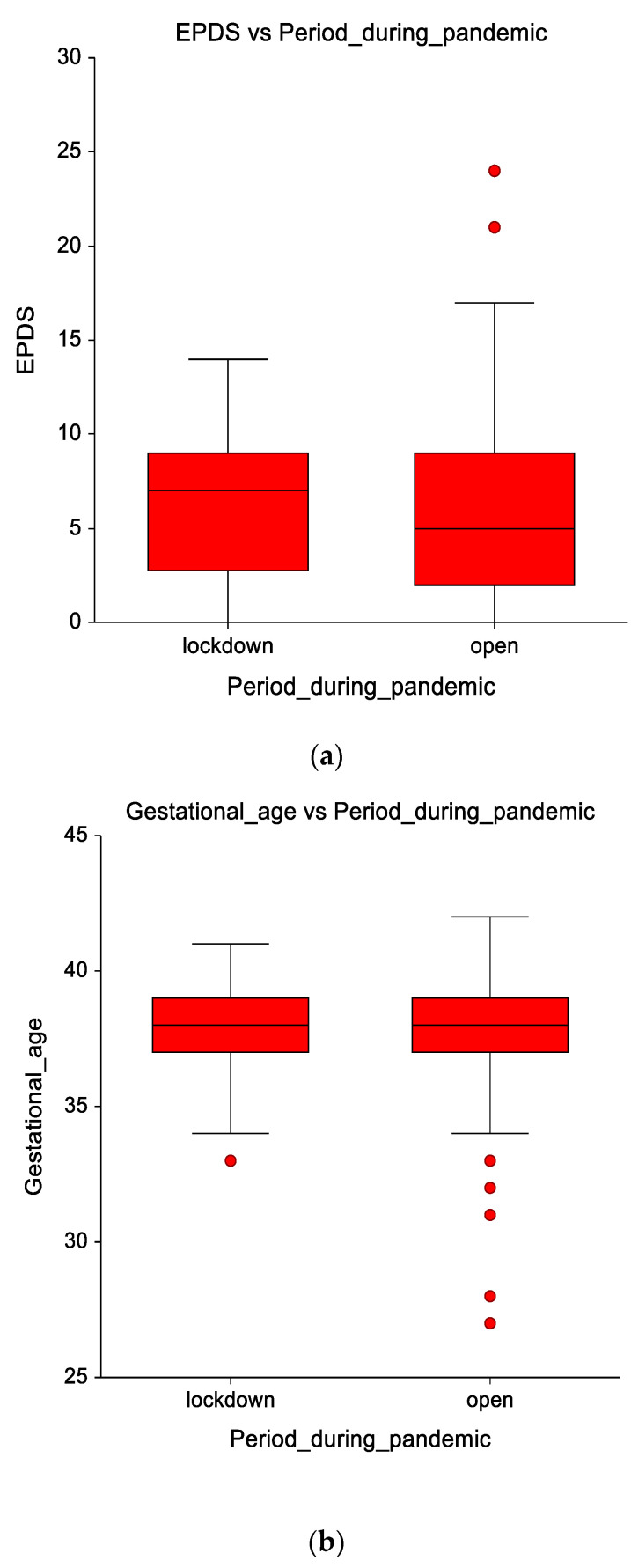
Comparison between the two study groups: lockdown and open of mean EPDS scores (**a**) and mean Gestational age (weeks) (**b**).

There was no statistically significant difference between groups: lockdown and open, regarding overall EPDS scores (*z* value 0.51) and gestational age (*z* value −0.60), as determined by the Mann–Whitney U test (Figure 1).

**Figure 2:**
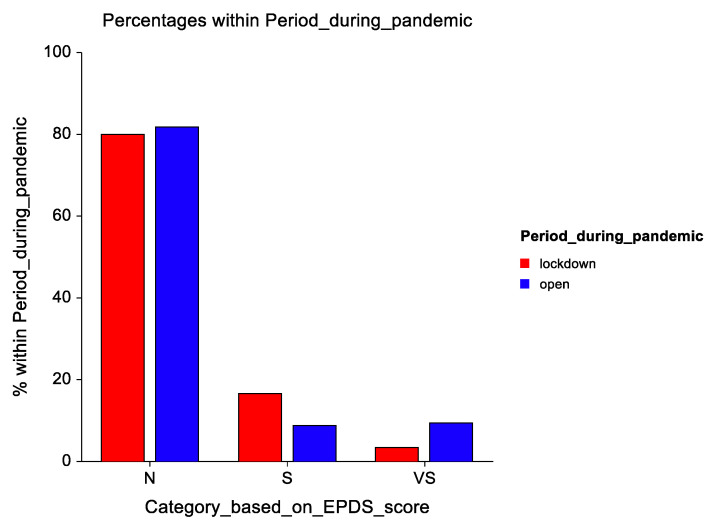
Distribution of patients based on EPDS scores and preestablished cut-off values.

The distribution of EPDS scores was made based on different score categories: normal (N), suspected of postpartum depression (S), and very suspicious with a referral (VS). During confinement, there were more patients in the S group and fewer in the vs. group: 5 versus 1, compared with the following period when more teenagers had more vs. scores than S scores: 16 versus 15 (Figure 2).

## 4. Discussion

Over time, postpartum depression has been extensively studied in adult mothers, but in the adolescent population, this mental disorder is still poorly characterized in standard psychiatry texts [12]. Comparing the 14–53% prevalence of depression in teenage mothers with 6.9–16.7% corresponding prevalence in adult mothers [12], the need for conventional healthcare procedures for prophylaxis, screening, diagnosis, and treatment of this disorder is reasonably justified. 

This study addresses the issue of postpartum depression in the adolescent population, which to our knowledge, is the first study of its kind to be developed in Romania. It also focuses on the impact of social restrictions related to the COVID-19 pandemic on postpartum depression. Results show that 2.99% of patients in early adolescence gave birth in our unit, making the “child with child” phenomena even more perturbing in our society. Additionally, most of the teenagers from our study (73.63%) came from rural areas. A proportion of 52.24% of overall patients graduated no more than five to eight years of school. Illiteracy rates extend from 21.89% of young girls who were unable to read to 22.39% who were unable to write. In a 2019 report, the Save the Children Association in Romania had similar findings on educational background and birthplace in the teenage mother population [13]. The relevance of these findings resides in their demonstrated association with depressive symptoms, according to Ribeiro et al., who stated that pregnant adolescents with four to seven years of education present 2.4 times more depressive symptoms compared to those with 11 or more years of education [14].

An alarming proportion of study participants (82.59%) confirmed living in concubinage with their husbands, and a similar percentage (85.07%) suggested that the pregnancy was planned. Since 66.67% of patients mentioned that giving birth to a baby before turning 20 years old was a tradition in their families, we consider this aspect relevant for the marital status of most study participants and their increased tendency towards early school dropout. Descriptive analysis of social and demographic variables for lockdown and open groups showed similar results to those presented for overall study participants. As stated by the United Nations Guidance document on Preventing adolescent pregnancy, a teenager’s pregnancy reflects the failure of those around her to protect her rights, including her right to protection from abuse and education that would provide her with opportunities and access to reproductive health information [15]. Therefore, irrelevant to the pandemic period, compromised basic teenager rights, and opportunities to reach for education and information should come as a priority to enact for all nations.

More adolescents who delivered during confinement felt stressed during the last year compared to those who delivered later in 2020 (16.67% versus 11.11%); on the other side, all patients from the lockdown group had family support during pregnancy, while in the other group family caring was assured in 94.15% of the cases. However, there were no statistically significant differences in the overall EPDS scores in the two study groups. This conclusion is in agreement with the multiple etiological models of postpartum depression that have been proposed in the literature [16]. 

Recent publications prove that restrictions related to social distance and measures of preventing communication with family and friends increase levels of stress, anxiety, and depression in pregnant women [17,18]. Having no reliable data on the impact of COVID-19 infection during pregnancy as well as facing the risk of exposure to the virus during regular prenatal medical follow-up are two factors converging to induce concern and stress during pregnancy. Salehi et al. concluded that the fear of COVID-19 during pregnancy could indirectly affect mental health by increasing the level of concerns during pregnancy [19]. Studies on epigenetic programming of central nervous system development related to stressful experiences during pregnancy reveal intriguing evidence on further transgenerational effects [20]. This explains why recently researchers have been focusing on perinatal depression; this affection is believed to be a factor involved in disruptive health development of infants—notably, novel findings show that maternal emotional neglect during her own childhood is associated with differences in the development of fronto-amygdala circuitry in the next generation as early as one month after birth [21]. 

Results in our multinomial regression showed that cigarette use had an incremental effect on the possibility of developing postpartum depression. This statement is in accordance with literature reports that identified low education, smoking, and stressful events as risk factors for depression [14]. Having a planned pregnancy and not being stressed were good predictors for significantly decreasing the likelihood of scoring EPDS points above normal. No other social and demographic factors were statistically reliable predictors of the model. Family support and obstetric variables did not manage to accurately predict overall EPDS scores. However, there is evidence suggesting that mutual influence of social and family support has a crucial role in antenatal depression; also, maternal age has been validated as a risk factor for antenatal depression, although in our study, no significant predictive value could be correlated [22,23]. Caution should be taken in interpreting the role of social and family support on the mother’s wellbeing since Beck has already described the interpersonal model of postpartum depression, explaining that there could also be a mismatch between the mother’s desired versus received support that could influence her depressive symptoms [16].

Since the pandemic debut, specialty organizations managed to implement multiple guidelines with specific indications for caring for pregnant women with COVID-19; the severe adverse effects of restricted postnatal care practices were also labeled [24,25]. Nevertheless, hidden maternal and fetal morbidity and documented public health consequences for the mother, child, and their family are serious concerns regarding adolescent mothers, even if tested negative for COVID-19 infection. Pregnant women asked to respond to a survey on the impact of COVID-19 on their mental status concluded that fear was the most prevalent feeling after the emergence of the pandemic [26]. 

The major study strength resides in the strict consideration of teenage mothers for assessing postpartum depression and the influence of the COVID-19 pandemic on their mental integrity; also, we were able to develop our study prospectively, during national confinement and the following period. This report has several limitations that we address: patient interviews occurred only once, during day 2 or 3 after birth, and no prenatal or further assessments were possible due to low addressability of patients to medical services; no COVID-19 positive patients responded to the interview since our unit was not COVID-19 dedicated; maternal comorbidities and neonatal factors that could influence maternal mental status were not objectified; it was a single-center study; comparing the study groups with patients who did not deliver under any social restrictions was not possible due to the prospective nature of this research. This study was designed to focus on young mothers only, and the patient-related data were collected during the pandemic when social restrictions were first established.

Interpreting pregnancy and birth both at the individual level and as an integrative part of the community dynamics can lead to the improvement of healthcare services and quality of care specifically. Pregnant teenagers are part of society, and their actions and behavior influence other members of the community as well. Assuring permanent support and physical and mental integrity during this transition to both motherhood and adult life rises as a commitment for obstetricians everywhere.

Our study addresses the problem of teenage pregnancy and its possible impact on maternal mental status during this fragile period in life; the COVID-19 pandemic represents a new distress generator that can further influence young mothers’ adaptability to changes in body and life. Caring for this population of mothers is a necessity since more and more adolescents succumb to temptation, and social pressure and specific medical guidelines are starting to become mandatory. Effects of the pandemic over the long term are assumptive to affect personal behavior and societal relations as well.

## 5. Conclusions

This study was able to demonstrate that although more adolescents who delivered in our unit during national confinement were stressed compared to those who delivered in the following period, this aspect did not interfere with postpartum depression screening scores. Having a planned pregnancy even during adolescence can serve as a protective factor for postpartum depression, and noxious behavior associated with cigarette use exposes teenage mothers to postnatal depression.

## Figures and Tables

**Table 1:** Baseline characteristics of sample groups.

	Overall *n* = 201, 100%		Lockdown *n* = 30, 14.92%		Open *n* = 171, 85.07%	
Age (years)	Count	Percent	Count	Percent	Count	Percent
10–14	6	2.99%	0	0.00%	6	3.51%
15–17	89	44.28%	14	46.67%	75	43.86%
18–19	106	52.74%	16	53.33%	90	52.63%
Residence						
Rural	148	73.63%	21	70.00%	127	74.27%
Urban	53	26.37%	9	30.00%	44	25.73%
Education *						
<1 year	19	9.45%	4	13.33%	15	8.77%
1–4 years	21	10.44%	2	6.66%	19	11.11%
5–8 years	105	52.24%	16	53.33%	89	52.05%
9–12 years	56	27.86%	8	26.67%	48	28.07%
Ability to write						
Yes	156	77.61%	25	83.33%	131	76.61%
No	45	22.39%	5	16.67%	40	23.39%
Ability to read						
Yes	157	78.11%	25	83.33%	132	77.19%
No	44	21.89%	5	16.67%	39	22.81%
Marital status						
Married	22	10.95%	2	6.67%	20	11.70%
Concubinage	166	82.59%	26	86.67%	140	81.87%
Alone	13	6.47%	2	6.67%	11	6.43%
Family support						
Yes	191	95.02%	30	100.00%	161	94.15%
No	10	4.98%	0	0.00%	10	5.85%
Family tradition						
Yes	134	66.67%	20	66.67%	114	66.67%
Unknown	15	7.46%	2	6.67%	13	7.60%
No	52	25.87%	8	26.67%	44	25.73%
Unintended pregnancy						
Yes	30	14.93%	4	13.33%	26	15.20%
No	171	85.07%	26	86.67%	145	84.80%
Stressed in the last year						
Yes	24	11.94%	5	16.67%	19	11.11%
No	177	88.06%	25	83.33%	152	88.89%

* expressed in the number of completed years of school.

**Table 2:** Summary of obstetric factors.

	Count	Mean	Std Dev	Min	Max
Parity					
Lockdown	30	1.53	0.86	1	4
Open	171	1.32	0.54	1	3
Abortions *					
Lockdown	30	0.3	0.59	0	2
Open	171	0.31	0.71	0	4
GA (weeks) **					
Lockdown	30	37.9	1.82	33	41
Open **	168	38.05	2.05	27	42

* induced and spontaneous, ** for three patients gestational age (GA) could not be established (deliveries without medical assistance).

**Table 3:** Multinomial logistic regression and coefficient significance tests.

Independent.	Regression	Standard	Wald		Odds
Variable	Coefficient	Error	*z*-Value	Wald	Ratio
	b(i)	Sb(i)	H0: β = 0	*p*-Value	Exp (b(i))
Education					
S	0.03657	0.07699	0.475	0.63482	103.724
VS	−0.09248	0.09986	−0.926	0.35438	0.91166
No of sexual partners					
S	−0.22069	0.32678	−0.675	0.49946	0.80197
VS	0.52650	0.35917	1.466	0.14268	169.299
Parity					
S	0.14212	0.38632	0.368	0.71295	115.272
VS	0.31132	0.63357	0.491	0.62316	136.522
No of cigarettes/day					
S	0.08030	0.03889	2.065	0.03894	108.362
VS	0.04515	0.05380	0.839	0.40135	104.619
Family support = “no”					
S	0.04996	0.93958	0.053	0.95759	105.123
VS	−0.61976	128.292	−0.483	0.62903	0.53807
Stressed over the last year = “no”					
S	−0.84933	0.64633	−1.314	0.18881	0.42770
VS	−254.094	0.69459	−3.658	0.00025	0.07879
Unintended pregnancy = “no”					
S	−137.665	0.51084	−2.695	0.00704	0.25242
VS	−222.566	0.73227	−3.039	0.00237	0.10800

## Data Availability

Study database is available upon request.
